# CADASIL mutations sensitize the brain to ischemia via spreading depolarizations and abnormal extracellular potassium homeostasis

**DOI:** 10.1172/JCI149759

**Published:** 2022-04-15

**Authors:** Fumiaki Oka, Jeong Hyun Lee, Izumi Yuzawa, Mei Li, Daniel von Bornstaedt, Katharina Eikermann-Haerter, Tao Qin, David Y. Chung, Homa Sadeghian, Jessica L. Seidel, Takahiko Imai, Doga Vuralli, Rosangela M. Platt, Mark T. Nelson, Anne Joutel, Sava Sakadzic, Cenk Ayata

**Affiliations:** 1 Neurovascular Research Unit, Department of Radiology, Massachusetts General Hospital, Harvard Medical School, Boston, Massachusetts, USA.; 2 Department of Neurosurgery, Yamaguchi Graduate School of Medicine, Yamaguchi, Japan.; 3 Therapeutics & Biotechnology Division, Korea Research Institute of Chemical Technology, Daejeon, South Korea.; 4 Department of Pharmacology, Larner College of Medicine, University of Vermont, Burlington, Vermont, USA.; 5 Division of Cardiovascular Sciences, University of Manchester, Manchester, United Kingdom.; 6 Institute of Psychiatry and Neuroscience of Paris — INSERM U1266, Université de Paris, GHU Paris Psychiatrie et Neurosciences, Hôpital Sainte Anne, Paris, France.; 7 Athinoula A. Martinos Center for Biomedical Imaging, Department of Radiology, Massachusetts General Hospital, Harvard Medical School, Charlestown, Massachusetts, USA.; 8 Stroke Service, Department of Neurology, Massachusetts General Hospital, Harvard Medical School, Boston, Massachusetts, USA.

**Keywords:** Neuroscience, Microcirculation, Potassium channels, Stroke

## Abstract

Cerebral autosomal dominant arteriopathy, subcortical infarcts, and leukoencephalopathy (CADASIL) is the most common monogenic form of small vessel disease characterized by migraine with aura, leukoaraiosis, strokes, and dementia. CADASIL mutations cause cerebrovascular dysfunction in both animal models and humans. Here, we showed that 2 different human CADASIL mutations (Notch3 R90C or R169C) worsen ischemic stroke outcomes in transgenic mice; this was explained by the higher blood flow threshold to maintain tissue viability compared with that in wild type (WT) mice. Both mutants developed larger infarcts and worse neurological deficits compared with WT mice, regardless of age or sex after filament middle cerebral artery occlusion. However, full-field laser speckle flowmetry during distal middle cerebral artery occlusion showed comparable perfusion deficits in mutants and their respective WT controls. Circle of Willis anatomy and pial collateralization also did not differ among the genotypes. In contrast, mutants had a higher cerebral blood flow threshold, below which infarction ensued, suggesting increased sensitivity of brain tissue to ischemia. Electrophysiological recordings revealed a 1.5- to 2-fold higher frequency of peri-infarct spreading depolarizations in CADASIL mutants. Higher extracellular K^+^ elevations during spreading depolarizations in the mutants implicated a defect in extracellular K^+^ clearance. Altogether, these data reveal a mechanism of enhanced vulnerability to ischemic injury linked to abnormal extracellular ion homeostasis and susceptibility to ischemic depolarizations in CADASIL.

## Introduction

Cerebral autosomal dominant arteriopathy, subcortical infarcts, and leukoencephalopathy (CADASIL) is the most common monogenic small vessel disease, characterized by frequent migraine attacks with aura, progressive white matter degeneration, and recurrent lacunar infarctions in young to middle-aged adults, culminating in vascular dementia (1). Notably, patients with CADASIL exhibit deficits in functional hyperemia and cerebrovascular reactivity to CO_2_ that can be present long before evidence of significant disability and cognitive deficits (2, 3). The disease is caused by highly stereotyped mutations in the NOTCH3 receptor, which is predominantly expressed in pericytes and smooth muscle cells of small vessels (4–6).

A number of genetic mouse models expressing Notch3 CADASIL mutations have been developed that recapitulate the CADASIL phenotype to various degrees (7, 8). Whereas most of these Notch3 mutant mouse models develop pathognomonic accumulation of Notch3^ECD^ and granular osmiophilic deposits in brain vessels, additional manifestations have been detected in a few models. For example, white matter lesions have been demonstrated only in TgNotch3^R169C^ mice, which overexpress a rat NOTCH3 protein under the endogenous Notch3 promoter with the Arg 169Cys mutation (9, 10). Moreover, impaired myogenic responses, neurovascular coupling during functional activation, and cerebral blood flow (CBF) autoregulation during blood pressure (BP) fluctuations have been reported only in TgNotch3^R169C^ mice and TgNotch3^R90C^ mice, which overexpress a human NOTCH3 under the smooth muscle SM22α reporter with the Arg90Cys mutation (9–13).

Spontaneous strokes have never been demonstrated in CADASIL mutants, likely due to the much shorter life span of mice than humans. However, given the evidence indicating cerebrovascular dysfunction in CADASIL mutants, one might anticipate larger ischemic infarcts linked to collateral insufficiency. We designed this study to test whether CADASIL mutations worsen focal ischemic outcomes in transgenic mice and to interrogate the hemodynamic mechanisms underlying this phenotype. Our findings indeed show a more severe stroke phenotype in CADASIL mutant mice but surprisingly implicate increased parenchymal sensitivity to ischemia, rather than cerebral hemodynamic dysfunction, as a key mechanism.

## Results

**Figure 1:**
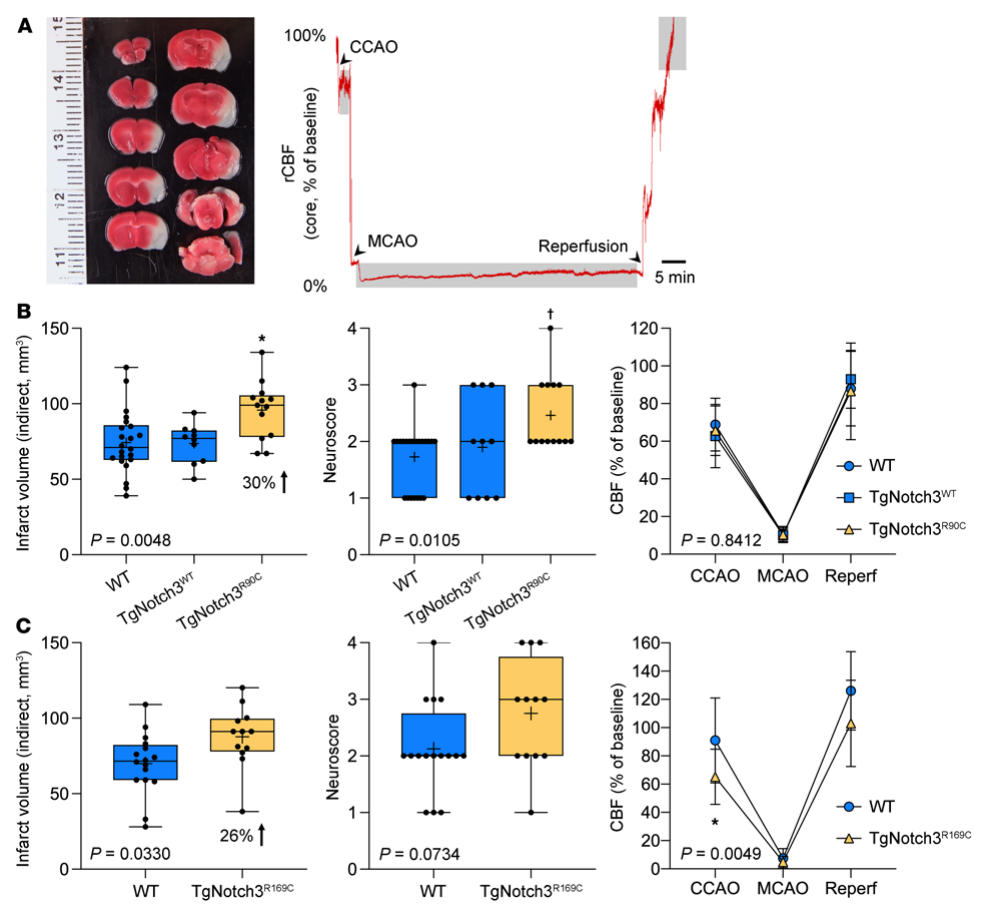
Filament middle cerebral artery occlusion in TgNotch3^R90C^ and TgNotch3^R169C^ cohorts. (**A**) Left: A representative image of 2,3,5-triphenyltetrazolium chloride–stained (TTC-stained) coronal sections 24 hours after transient filament middle cerebral artery occlusion (fMCAO). Infarct can be seen as nonstained tissue involving MCA territory. Right: Representative laser Doppler flowmetry (LDF) tracing shows decrease in regional cerebral blood flow (CBF) after common carotid artery occlusion (CCAO) followed by MCAO and reperfusion. Shaded segments indicate where CBF was measured relative to baseline. (**B**) Infarct volume (indirect method), neurological deficit score, and CBF after CCAO, during MCAO, and after reperfusion in the entire TgNotch3^R90C^ cohort (all ages pooled). In addition to nontransgenic WT mice, transgenic mice overexpressing the human WT Notch 3 (TgNotch3^WT^ mice) were used to control for overexpression in TgNotch3^R90C^ mice. One-way ANOVA followed by Tukey’s multiple comparisons for infarct volume and neurological deficit score (*P = 0.0035 vs. WT, *P = 0.0074 vs. TgNotch3^WT^; †*P =* 0.0039 vs. WT, †*P =* 0.0372 vs. TgNotch3^WT^) and 2-way ANOVA for repeated measures followed by Šidák’s multiple comparisons for CBF. ANOVA *P* values are also shown on each panel. Sample sizes are provided in Table 4. (**C**) Infarct volume (indirect method), neurological deficit score, and CBF after CCAO, during MCAO, and after reperfusion in the entire TgNotch3^R169C^ cohort (all ages pooled, Table 4). Unpaired *t* test for infarct volume and neurological deficit score, and two-way ANOVA for repeated measures followed by Šidák’s multiple comparisons for CBF (*P = 0.0326, TgNotch3^R169C^ vs. WT). ANOVA *P* values are also shown on each panel. Mean ± SD. Sample sizes are provided in Table 4.

We first examined the effect of Notch3^R90C^ mutation on the outcome of filament middle cerebral artery occlusion (fMCAO) (Figure 1A) in WT, TgNotch3^WT^, and TgNotch3^R90C^ mice (male, aged 3–22 months old). To account for the effect of age, we performed multivariable regression (independent variables: genotype and age; dependent variables: infarct volume, neurological deficit scores, swelling volume, and CBF after common carotid occlusion [CCAO], during middle cerebral artery occlusion [MCAO], or after reperfusion). Regression models predicted infarct volume, neurological deficit score, swelling volume, and CBF after reperfusion but not CBF after CCAO or during MCAO (Table 1; see Supplemental Figure 1 for individual data points; supplemental material available online with this article; https://doi.org/10.1172/JCI149759DS1). Genotype, but not age, significantly contributed to the infarct volume. TgNotch3^R90C^ mice developed larger infarcts than WT mice. Genotype and age both contributed to the neurological deficit score. TgNotch3^R90C^ mice developed worse deficit scores than WT mice, while age showed a weak inverse relationship. Interestingly, age, but not genotype, contributed to the swelling volume, which decreased with age. None of the independent variables contributed to CBF after CCAO and during MCAO, and only age significantly contributed to the CBF after reperfusion. After confirming the independent contribution of genotype to outcome endpoints using multivariable analysis as above, all ages were pooled for genotype comparisons (Figure 1B). In the pooled cohort, TgNotch3^R90C^ mice but not TgNotch3^WT^ mice had larger infarcts and worse neurological deficit scores compared with the WT mice. Importantly, TgNotch3^WT^ mice, which overexpress normal Notch3 gene, did not differ from WT mice in any endpoint, indicating that the phenotype was not simply due to Notch3 overexpression. These data suggested that Notch3^R90C^ mutation worsened focal cerebral ischemic outcomes but did not support a hemodynamic mechanism.

To strengthen the causal link between CADASIL mutations and focal ischemic outcomes, we examined another CADASIL mouse model expressing the Notch3^R169C^ mutation (male and female, ages ~3–7 months old). A multivariable regression (independent variables: genotype and age) model significantly predicted the infarct volume but not the other dependent variables (Table 2). Genotype, but not age or sex, contributed significantly to the infarct volume. In the pooled cohort, infarcts were significantly larger in the TgNotch3^R169C^ mutant mice (Figure 1C). Although CBF after CCAO appeared lower in the mutant mice, CBF during MCAO and after reperfusion did not differ from that in WT mice (Figure 1C), once again not supporting a hemodynamic mechanism for larger infarcts.

**Figure 2:**
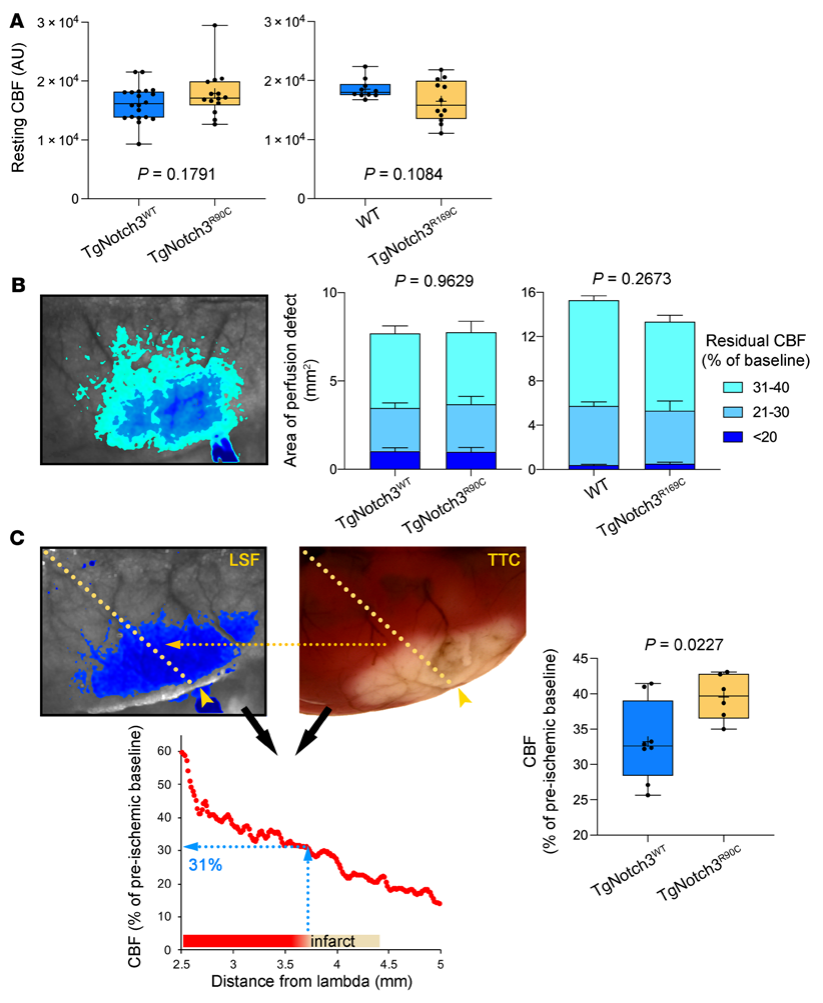
Distal middle cerebral artery occlusion in TgNotch3^R90C^ and TgNotch3^R169C^ cohorts. (**A**) Resting CBF calculated using laser speckle contrast inverse correlation time values before distal middle cerebral artery occlusion (dMCAO) did not differ between CADASIL mutant mice and controls. Sample sizes are provided in Table 4. Student’s *t* test. (**B**) A representative laser speckle contrast image taken 60 minutes after dMCAO shows regions with severe (residual CBF <20%), moderate (21%–30%), and mild (31%–40%) CBF deficit. Composite bar graphs show the areas of severe, moderate, and mild CBF deficit in TgNotch3^R90C^ and TgNotch3^R169C^ mice and their respective controls (TgNotch3^WT^ and WT) 60 minutes after dMCAO (all ages pooled). Two-way ANOVA for repeated measures. *P* values on each panel are those of main ANOVA. CBF deficit area is shown as mean ± SEM. (**C**) A representative laser speckle contrast image showing the perfusion defect during dMCAO (left) and 2,3,5-triphenyltetrazolium chloride–stained (TTC-stained) brain showing the infarct in the same brain 48 hours later (right). Images were spatially coregistered using surface landmarks. A line profile was drawn between lambda and the clip occluding the middle cerebral artery (yellow arrowhead). For each animal, CBF was plotted along the line profile as a function of distance from lambda using laser speckle images (bottom). The CBF at the infarct edge was determined (blue dotted lines), representing the CBF threshold for viability, below which tissue infarcted in each mouse. The average viability threshold was significantly higher in TgNotch3^R90C^ vs. TgNotch3^WT^ mice (all ages pooled). Unpaired *t* test.

**Figure 3:**
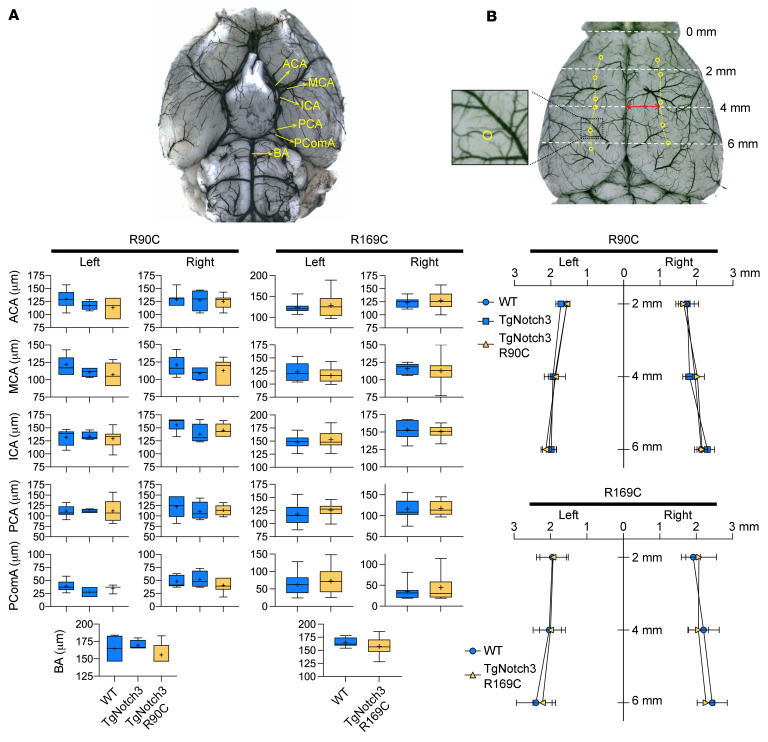
Cerebrovascular anatomy in Notch3^R90C^ and Notch3^R169C^ cohorts. Representative (**A**) ventral and (**B**) dorsal views show the circle of Wills anatomy and pial arterial anastomoses between middle and anterior cerebral arteries. Circles on the dorsal surface in **B** indicate the pial anastomoses analyzed for their number and distance to midline. ACA, anterior cerebral artery; BA, basilar artery; ICA, internal carotid artery; MCA, middle cerebral artery; PCA, posterior cerebral artery; PComA, posterior communicating artery. The sample size is 39 in total and details are provided in Table 4. One-way ANOVA, 2-way ANOVA, or unpaired *t* test. Panel **A** shows diameters of major arteries in the circle of Willis. Panel **B** shows the distance of pial collaterals from midline. Mean ± SD.

Since laser Doppler flowmetry (LDF) provides a single point relative measure of CBF and cannot ascertain the differences in the overall area of the perfusion defect or resting CBF, we next used full-field laser speckle flowmetry (LSF) to better estimate resting CBF and quantify the acute perfusion defect with high spatiotemporal resolution during distal MCAO (dMCAO). The multivariable model accounting for genotype, age, sex, and arterial BP did not predict resting CBF prior to dMCAO or the area of perfusion defect after dMCAO (Table 3). Neither resting CBF (Figure 2A) nor the area of perfusion defect (Figure 2B) differed between TgNotch3^R90C^ and TgNotch3^R169C^ CADASIL mutant mice and their respective controls. These data confirmed LDF findings in fMCAO and indicated that Notch3 mutations do not impair collateral flow during focal cerebral arterial occlusions. Moreover, examination of cerebrovascular anatomy also did not reveal a difference between animals with CADASIL mutations and their respective WT controls in the diameter of major intracranial arteries and the number and location of pial anastomoses (Figure 3).

An alternative mechanism for larger infarcts in animals with CADASIL mutations was higher tissue sensitivity to ischemia. Therefore, we next determined the critical tissue CBF below which infarction ensued (i.e., viability threshold) in TgNotch3^WT^ and TgNotch3^R90C^ mice. For this, we calculated the regional CBF at the infarct margin by spatially coregistering the LSF map during dMCAO with the dorsal view of the infarct 48 hours later (Figure 2C). We found higher viability thresholds in the CADASIL mutant, indicating that the cortex is more vulnerable to ischemic injury and required a much higher residual CBF to survive.

**Figure 4:**
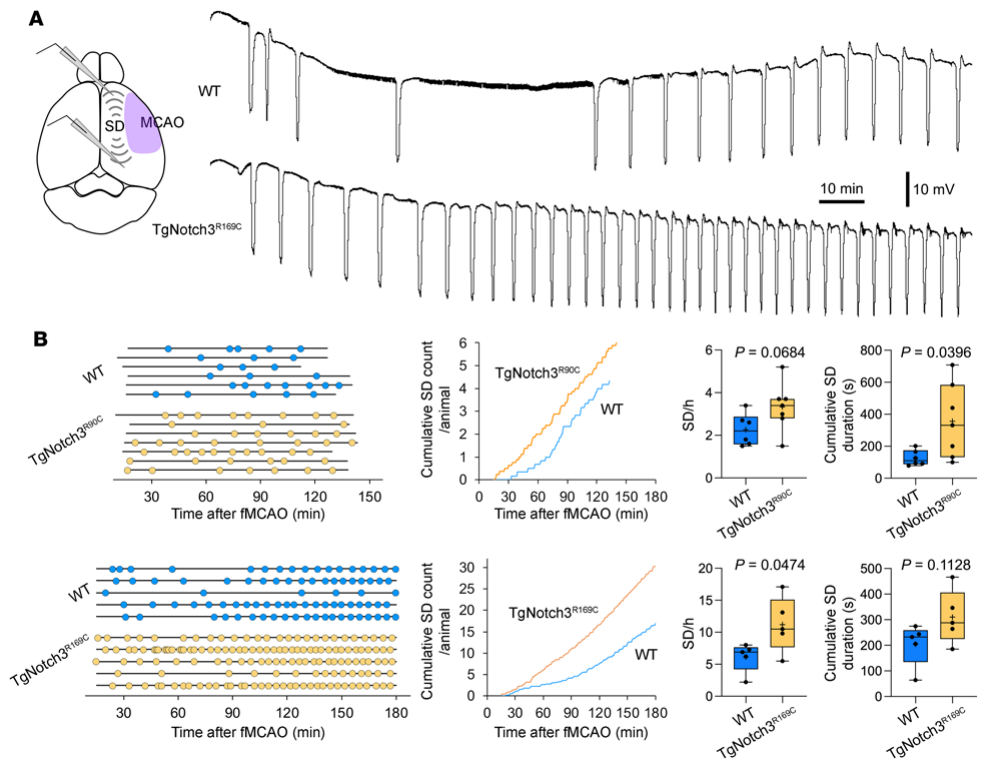
Peri-infarct spreading depolarization during filament middle cerebral artery occlusion in Notch3^R90C^ and Notch3^R169C^ cohorts. (**A**) Representative extracellular DC potential recordings from peri-infarct cortex showing higher frequency of peri-infarct spreading depolarizations (SDs) in TgNotch3^R169C^ mice compared with WT mice after filament middle cerebral artery occlusion (fMCAO). Experimental setup shows intracortical glass micropipettes placed outside the ischemic core (purple area) to detect SDs. (**B**) Left: Experimental timelines showing the time of onset and end of recordings in each mouse, and time of occurrence of SDs (round symbols) in WT and TgNotch3^R90C^ or TgNotch3^R169C^ mice. Middle: Pooled cumulative SD numbers per animal over time after fMCAO. Right: The frequency of SDs and cumulative SD duration in WT and TgNotch3^R90C^ mice or TgNotch3^R169C^ mice. Unpaired *t* test. Sample sizes are provided in Table 4.

We have previously shown that CADASIL mutations increase susceptibility to spreading depolarizations (SD), consistent with the migraine with aura phenotype in this disease (14). In separate studies, we have also shown that higher susceptibility to SD leads to worse stroke outcomes (15) and vice versa (16). Therefore, we next examined whether CADASIL mutations augment peri-infarct SDs (PIDs), which are known to exacerbate the supply demand mismatch in penumbra and promote acute infarct growth (17). Using intracortical microelectrodes placed in the peri-infarct cortex during fMCAO (Figure 4A), we found increased frequency or cumulative duration of PIDs in both TgNotch3^R90C^ and TgNotch3^R169C^ mutant mice compared with their respective controls (Figure 4B). Because plasma glucose is an important determinant of SD occurrence, we measured this in a separate cohort (*n =* 6 WT and TgNotch3^R169C^ each, male/female = 1, age 14–17 weeks) and found comparable blood glucose levels (234 ± 15 mg/dL and 217 ± 16 mg/dL, mean ± SEM, in WT and TgNotch3^R169C^, respectively; *P =* 0.457, unpaired *t* test).

**Figure 5:**
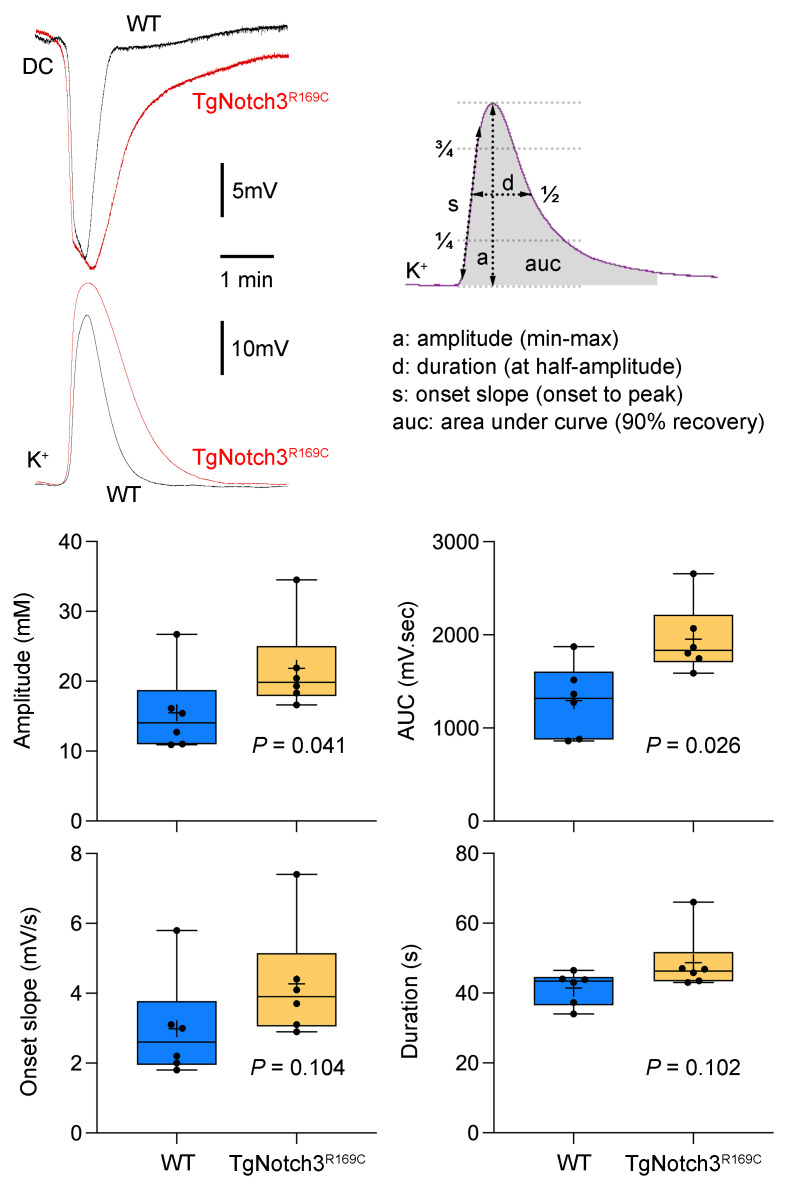
Extracellular K^+^ rise during spreading depolarizations in nonischemic cortex. Representative extracellular DC potential and K^+^-sensitive electrode tracings show the measurement of the amplitude (a), area under curve (auc), onset slope (s), and duration (d) of the K^+^ surge during an SD. Graphs show these measurements. Unpaired *t* test for repeated measures. Sample sizes are provided in Table 4. Mean ± standard error.

Given that Notch3 is predominantly expressed in pericytes and smooth muscle cells of small vessels, increased PID was an intriguing finding, implicating a defect in the neurovascular interface affecting ion homeostasis. To test this possibility, we examined extracellular K^+^ concentration [K^+^]_e_ dynamics using ion-selective electrodes. Because of the high spatiotemporal variability in [K^+^]_e_ in ischemic penumbra both within and among animals, we studied [K^+^]_e_ changes during SD in the nonischemic cortex. We found that the characteristic [K^+^]_e_ surge during SDs was larger in TgNotch3^R169C^ mice compared with WT mice (Figure 5). These data confirmed abnormal extracellular K^+^ handling during intense depolarization states.

## Discussion

In this study, we show enlarged focal cerebral ischemic infarcts in 2 independent transgenic mouse models expressing typical human CADASIL mutations Notch3^R90C^ or Notch3^R169C^. Contrary to our expectations, however, CADASIL mutations did not affect residual tissue perfusion, effectively ruling out cerebrovascular dysfunction and collateral insufficiency as an explanation of the larger infarcts. This was particularly surprising given the almost exclusive expression of Notch3 in smooth muscle cells and pericytes in adult brain, and impaired neurovascular coupling, autoregulation, and myogenic responses established in these CADASIL mutants (9–13, 18–20). The fact that aging did not affect the outcomes in our study also suggested that mechanisms were unrelated to CBF, since the cerebrovascular phenotype in CADASIL mutants progresses with aging.

Instead, the elevated viability threshold for CBF in CADASIL mutants suggested that the brain tissue required higher amounts of CBF to survive. This was reminiscent of familial hemiplegic migraine type 1–knockin (FHM1-knockin) mice (15). Indeed, both FHM1 (21) and CADASIL (14) mutations increase susceptibility to SD, the electrophysiological phenomenon underlying migraine aura (22, 23). Enhanced susceptibility to SD is the likely mechanism of frequent and often severe migraine with aura phenotype in both FHM1 and CADASIL (24, 25). Moreover, FHM1 mice also develop very high numbers of PIDs after fMCAO (15), and data herein revealed a similar phenotype associated with two different human CADASIL mutations.

SDs are intense pandepolarization waves that slowly propagate (~2–5 mm/min) in contiguous brain tissue, accompanied by loss of transmembrane ion gradients and a massive K^+^ efflux that elevates [K^+^]_e_ by approximately 10-fold for up to one minute (17). Our data suggest that the immediate mechanism of enlarged infarcts in the CADASIL mutant is enhanced susceptibility to SDs, which are widely believed to worsen the supply-demand mismatch in ischemic penumbra (26–31). Moreover, intracortical recordings showed a larger extracellular K^+^ ([K^+^]_e_) surge with steeper onset during SD in CADASIL mutants, suggesting abnormal [K^+^]_e_ buffering. This could explain the enhanced susceptibility to SD in CADASIL mutant mice, as well as the frequent and severe migraine with aura phenotype in patients with CADASIL (14, 32, 33). Recent data suggesting that an impaired K^+^ clearance increases the frequency of spontaneous neuronal glutamatergic plumes and predisposes to SD events may support this hypothesis (34).

**Figure 6:**
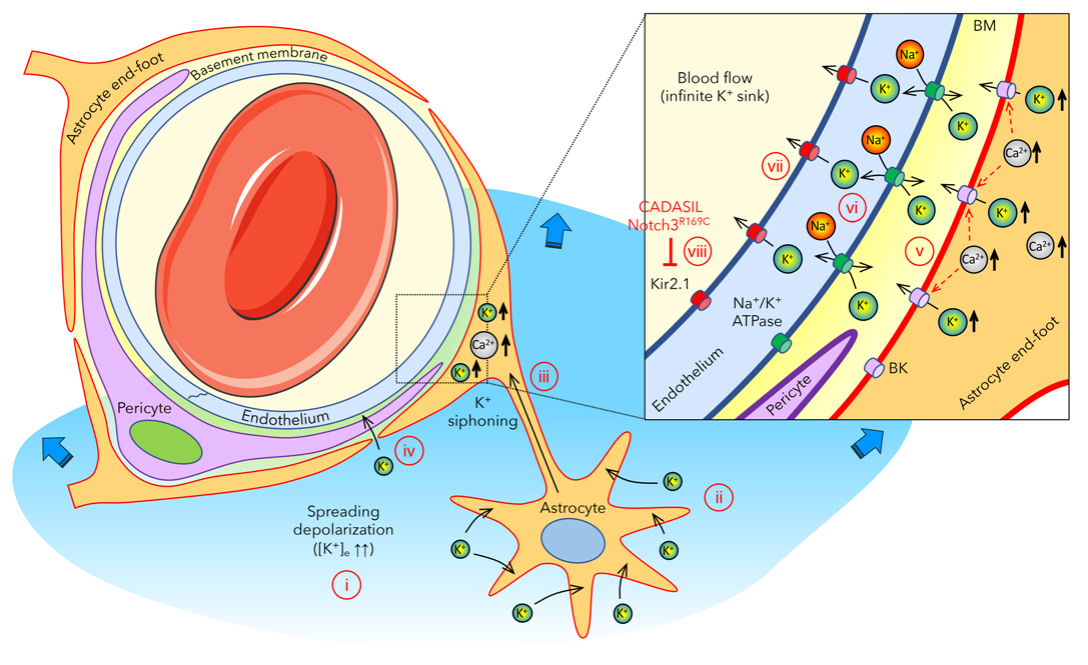
Proposed gliovascular mechanism of extracellular K^+^ regulation when local buffering mechanisms are exceeded during spreading depolarizations. (i) Upon intense depolarization states, such as anoxic or spreading depolarization, extracellular K^+^ concentration ([K^+^]_e_) can rise above the 10–12 mM ceiling. (b) Astrocytes play a major role in regulating [K^+^]_e_ via rapid uptake and spatial buffering through the astrocytic syncytium. (c) Astrocytes send their end feet, almost completely encasing the cerebral vasculature, including the capillary bed, providing a route for gliovascular K^+^ siphoning. (d) The massive rise in [K^+^]_e_ during an SD might also facilitate direct entry of K^+^ into the perivascular space to reach the capillary endothelium. (e) Astrocyte end feet have high K^+^ conductance, in part, due to BK channels activated by intracellular Ca^2+^ elevations, such as those observed during SD, and release large amounts of K^+^ into the tight perivascular space. (f) This perivascular K^+^ is then taken up by the endothelial Na^+^/K^+^-ATPase, which is densely — and asymmetrically — localized on the abluminal membranes. (g) Endothelial cells then release the K^+^ into the blood stream via channels and/or pumps on the luminal membrane, including K_ir_2.1, which is known to be activated by elevated perivascular [K^+^]_e_. (h) Notch3^R169C^ mutation is associated with impaired endothelial K_ir_2.1 channel function, linking CADASIL to impaired vascular K^+^ clearance.

The mechanism of abnormal [K^+^]_e_ buffering, however, is yet unknown, although evidence points toward a vascular mechanism. In the adult mammalian brain, Notch3 is exclusively expressed in the vasculature (i.e., pericytes and smooth muscle cells), and CADASIL mutations have been associated with vascular dysfunction (9, 10, 13, 18). By contrast, there has been no evidence to suggest a defect in astrocytic or neuronal K^+^ uptake in CADASIL. Therefore, we here postulate a model in which a vascular defect in CADASIL underlies the abnormal [K^+^]_e_ buffering in the brain (Figure 6). Astrocytes play a major role in regulating [K^+^]_e_ via rapid uptake and spatial buffering through the astrocytic syncytium (35, 36). Astrocytes also send their end feet, which almost completely encase the cerebral vasculature, including the capillary bed. The density and sheer surface area of the capillary bed makes it ideally positioned to expel excess [K^+^]_e_ into the blood stream. The latter would serve as an infinite (i.e., nonsaturable) sink. Hence, vascular clearance may be a fundamental mechanism when local buffering mechanisms are exceeded during intense depolarizations, such as SD, with massive elevations in [K^+^]_e_. Such gliovascular K^+^ siphoning has previously been proposed (37–39) based on observations in the closely related retina, where Müller glia siphon local K^+^_e_ into the vitreous (40). In the central nervous systems, astrocyte end feet encasing the vasculature indeed have an unusually high K^+^ conductance (37). This is, in part, due to the presence of large conductance BK channels activated by intracellular Ca^2+^ ([Ca^2+^]_i_) elevations (41, 42), such as those observed during SD (43, 44). Once activated, BK channels release large amounts of K^+^ into the tight perivascular space, especially in the setting of brain injury (45). In addition, the massive rise in [K^+^]_e_ to more than 20 mM during an SD might facilitate direct passive diffusion of K^+^ into the perivascular space to reach the capillary endothelium. This perivascular K^+^ is then taken up by endothelial Na^+^/K^+^-ATPase, which is densely — and asymmetrically — localized on the abluminal membranes (46). Endothelial cells must then release the K^+^ into the blood stream via channels and/or pumps on the luminal membrane, including K_ir_2.1 (47), which is known to be activated by elevated perivascular [K^+^]_e_ (41). Indeed, Notch3^R169C^ mutation has recently been associated with impaired endothelial K_ir_2.1 channel function due to ATP or phosphatidylinositol 4,5-bisphosphate (PIP_2_) shortage (48), providing at least one mechanism by which CADASIL mutations may interfere with vascular K^+^ clearance (13, 49). Other abnormalities, such as reduced endothelial expression or activity of Na^+^/K^+^-ATPase, remain to be tested.

The implications of our data for the spontaneously occurring lacunar strokes in patients with CADASIL are unclear, given that our model involved induced, rather than spontaneous, occlusion of a cortical, rather than subcortical, artery. Although CADASIL is a small vessel disease, we can nevertheless infer that the effect of ischemia, no matter how small the occluded artery, will be worse in CADASIL brains due to abnormal K^+^ handling and the propensity to develop ischemic depolarizations. This brings forth a mechanism by which CADASIL mutations can perturb the homeostasis and directly affect excitability early during the disease process, independent of the presumed mechanisms related to vasomotor dysfunction. Whether and how these mechanisms are linked to the characteristic deep white matter disease in CADASIL is unclear, especially because SDs are typically limited to gray matter structures. Future work using animal models of white matter ischemia might shed some light on this question.

## Methods

A total of 218 male and female mice, aged approximately 2–22 months, were used (Table 4), including 2 different mutant mouse models expressing 2 distinct typical CADASIL mutations (TgNotch3^R90C^ and TgNotch3^R169C^ mice; refs. 9, 12). In addition to WT littermates, transgenic mice overexpressing the human WT Notch 3 (TgNotch3^WT^ mice) were used as controls for TgNotch3^R90C^ mice (50). To avoid redundancy and unnecessary use of experimental animals, selected experiments were performed in only 1 mutant model or comparisons were made to a single control group.

### General surgery.

Mice were anesthetized with isoflurane (5% induction, 1% maintenance, in 70% N_2_/30% O_2_). When indicated, arterial pH, pO_2_, pCO_2_, and BP were measured via a femoral artery catheter (Supplemental Table 1). Rectal temperature was kept at 37°C using a thermostatic heating pad. In survival experiments, mice were placed in a temperature-controlled incubator with easy access to food and water. We closely monitored the animals for up to 48 hours following the procedure and recovery from anesthesia for signs of pain and discomfort.

### fMCAO.

Spontaneously breathing mice were anesthetized with isoflurane as above, a nylon monofilament was inserted into the right internal carotid artery via the external carotid artery, and MCA was occluded for 60 minutes (Notch3^R90C^) or 45 minutes (Notch3^R169C^), followed by reperfusion. These occlusion times were chosen based on prior experience with outcomes in respective genetic background strains. During surgery, CBF was monitored by LDF over the right temporal bone corresponding to ischemic core. Neurological outcomes were scored 24 hours after reperfusion using a 5-point scale (0, normal; 1, fore paw monoparesis; 2, circling to left; 3, falling to left; 4, no spontaneous walking and depressed consciousness). Infarct volume was then calculated by integrating the infarct area in ten 2,3,5-triphenyltetrazolium chloride–stained (TTC–stained) coronal sections at a thickness of 1 mm and subtracting the volume of ipsilateral noninfarcted tissue from the contralateral hemisphere (i.e., indirect). Ischemic swelling was calculated by subtracting the volume of contralateral hemisphere from the ipsilateral hemisphere.

### dMCAO.

Mice were intubated and mechanically ventilated (SAR-830 ventilator, CWE), except to minimize morbidity in cohorts where outcome was assessed 48 hours later. Mice were placed on a stereotaxic frame and scalp reflected via a midline incision. The skull overlying the right hemisphere was covered with a thin layer of mineral oil to prevent drying and enhance transparency. A temporal burr hole (2 mm diameter) was drilled above the zygomatic arch, and MCA was occluded using a microvascular clip for 60 minutes as described in detail previously (15, 28, 51). Absence of inadvertent mechanical induction of SD during drilling was confirmed by LSF (see below). Of note, dMCAO experiments to examine the perfusion defect (see below) were performed using endotracheal intubation and femoral artery cannulation and, thus, were terminal, precluding infarct volume determination.

### LSF.

LSF was performed using a near-infrared laser diode (785 nm, 75 mW) and a CCD camera (Cohu 4600, 640 × 480 pixels). Raw speckle frames were continuously acquired at 2.5 Hz. The laser speckle contrast inverse correlation time values (1/τ_c_) reflect resting CBF in arbitrary units, validated using the [^14^C]iodoamphetamine technique (51), which allows comparisons to be made among groups of animals imaged using identical surgical preparation and optical settings (51–53). CBF changes were also calculated for each pixel relative to the preischemic baseline, and the area of cortex with residual CBF of less than 20%, 21%–30%, or 31%–40% was determined by thresholding. In addition, the CBF threshold for tissue viability was estimated by superimposing the images of average CBF during MCAO and TTC-stained whole brain 48 hours later, with the same field of view and angle, as previously described (15). These brains were TTC-stained *topically* (rather than coronal slices) to preserve cortical contiguity. Although topical TTC staining yields a well demarcated infarct on the dorsal cortical surface, the dye does not penetrate full cortical thickness, precluding reliable infarct volume determinations.

### Electrophysiological recordings during fMCAO.

After fMCAO in a separate cohort, mice were placed in a stereotaxic frame and burr holes were drilled over the right hemisphere under saline cooling at (from bregma) (a) posterior 3.5 mm, lateral 2.0 mm (occipital, 0.5 mm diameter for electrode 1) and (b) anterior 1.0 mm, lateral 2.0 mm (frontal, 0.5 mm diameter for electrode 2). Dura was kept intact to minimize trauma to the animal. These coordinates were selected to be outside the ischemic core to detect PIDs. The steady (DC) potential and electrocorticogram were recorded with glass micropipettes filled with 154 mM NaCl placed 300 μm below pia (Axoprobe-1A, Axon Instruments). The Ag/AgCl reference electrode was placed subcutaneously in the neck. Monitoring started within 20 minutes after the onset of ischemia and continued for up to 3 hours. Frequency of PIDs (per hour of recording) and cumulative PID duration measured at half maximal amplitude were calculated.

### [K^+^]_e_.

[K^+^]_e_ were measured in 19-week-old male WT and TgNotch3^R169C^ mice using K^+^-selective electrodes prepared as previously described with some modifications (54). Double-barreled capillaries were pulled with a micropipette puller (Sutter Instruments). The ion-sensitive barrel was filled with potassium chloride (100 mM, in 154 mM NaCl), and the tip (2–3 μM) was silanized and filled with a K^+^-sensitive resin (Liquid Ion Exchanger IE190, WPI). The reference barrel was filled with sodium chloride (154 mM). Before each experiment the K^+^-sensitive microelectrodes were calibrated in K^+^ solution. Three consecutive SDs were induced every 15 minutes by topical application of 1 mm cotton ball soaked in 300 mM KCl onto the right occipital cortex. Measurements of [K^+^]_e_ were carried out in somatosensory cortex (2 mm lateral and 2 mm caudal to Bregma), more than 2 mm away from the SD induction site, at a depth of 300 μm.

### Anatomic analysis of the circle of Willis and pial collaterals.

Mice were intracardiac perfused with carbon black. The diameter of the major cerebral arteries; the number of pial arterial anastomoses among the anterior, posterior, and middle cerebral artery branches; and their distance from midline were determined on photographs taken after careful removal of the brain.

### Blood glucose.

Blood glucose was measured in a separate cohort of mice via tail snip under brief anesthesia (2.5% induction, 1.5%–2% maintenance in 70% N2O/30% O_2_), using ONETOUCH Ultra test strip and Ultra 2 meter (LifeScan IP Holdings).

### Statistics.

Data were analyzed using multiple linear regression, 1- or 2-way ANOVA, 2-tailed *t* test, or Mann-Whitney *U* test, as indicated by the data structure. All statistical tests are indicated in table or figure legends where data are presented. Normality was checked by Shapiro-Wilk test. Nonparametric tests were used for data sets that failed normality. Initial sample sizes were selected empirically to achieve 80% power to detect a 30% effect size based on an assumed standard deviation of 30% of the mean (α = 0.05). If the initial observed coefficient of variation deviated from assumed values, sample size calculations were revised accordingly. Variations in availability of different genotypes in breeding colonies also factored in final sample sizes. Although we planned to use both male and female mice as well as young and aged mice, we did not intend to select sample sizes powered to detect sexual dimorphism or effect of aging. Nevertheless, multivariable analyses accounted for their contribution to outcomes. All sample sizes, mortality, and exclusions due to technical failures are shown in Table 4. Absence of a treatment arm obviated randomization. All experiments were carried out blinded and confirmatory genotyping was done. *P* values of less than 0.05 were considered significant.

### Study approval.

All experimental procedures were carried out in accordance with the ARRIVE guidelines and the *Guide for the Care and Use of Laboratory Animals* (National Academies Press, 1996) and were approved by the institutional review board (the Massachusetts General Hospital Institutional Animal Care and Use Committee, Boston, MA, USA).

## Author contributions

FO performed the experiments, analyzed the data, and wrote the manuscript. JHL, IY, ML, DVB, TQ, DYC, HS, JLS, DV, TI, and RMP performed the experiments and analyzed the data. KEH, MTN, AJ, and SS provided intellectual input and revised the manuscript. CA designed the study, analyzed the data, and wrote the manuscript.

## Supplementary Material

Supplemental data

## Figures and Tables

**Table 1:**
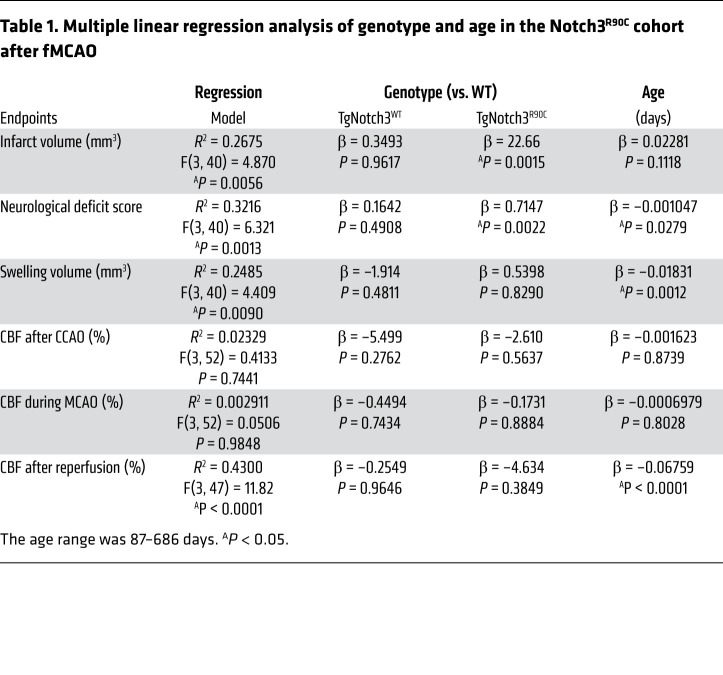
Multiple linear regression analysis of genotype and age in the Notch3^R90C^ cohort after fMCAO

**Table 2:**
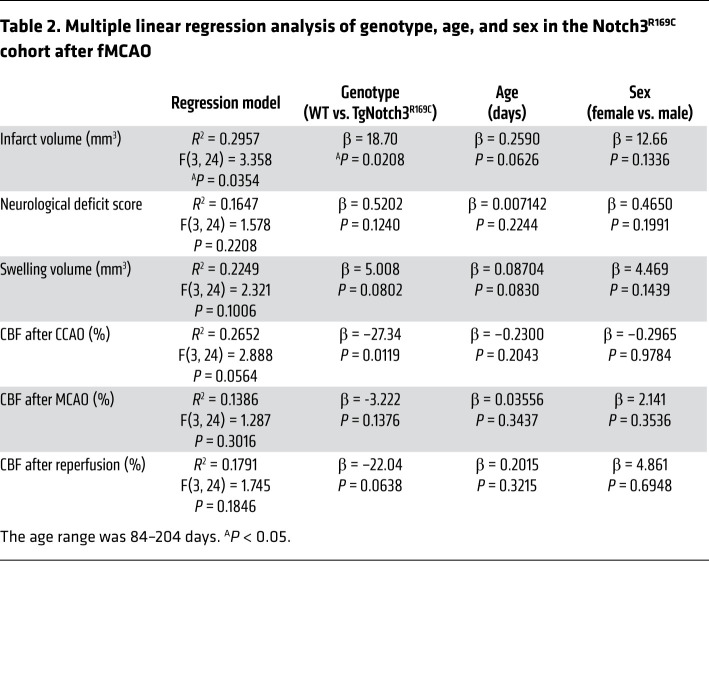
Multiple linear regression analysis of genotype, age, and sex in the Notch3^R169C^ cohort after fMCAO

**Table 3:**
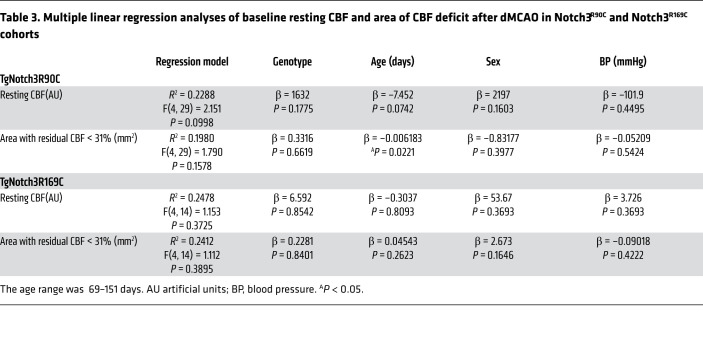
Multiple linear regression analyses of baseline resting CBF and area of CBF deficit after dMCAO in Notch3^R90C^ and Notch3^R169C^ cohorts

**Table 4:**
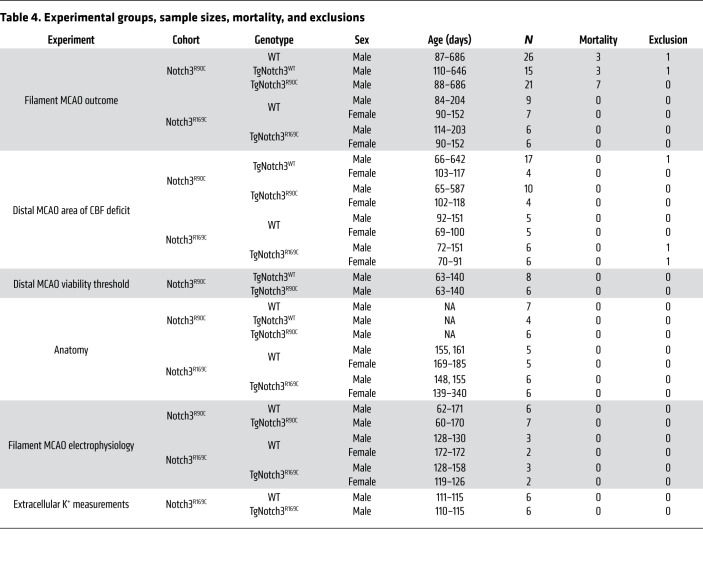
Experimental groups, sample sizes, mortality, and exclusions

## Version 1 02/24/2022

In-Press Preview

## Version 2 04/15/2022

Electronic publication
