# Supplementary material for: CADASIL mutations sensitize the brain to ischemia via spreading depolarizations and abnormal extracellular potassium homeostasis
Source: J Clin Invest. 2022 Apr 15;132(8):e149759. doi: 10.1172/JCI149759 (PMC9012276; doi:10.1172/JCI149759)
Supplement: Supplemental data [file jci-132-149759-s061.pdf]

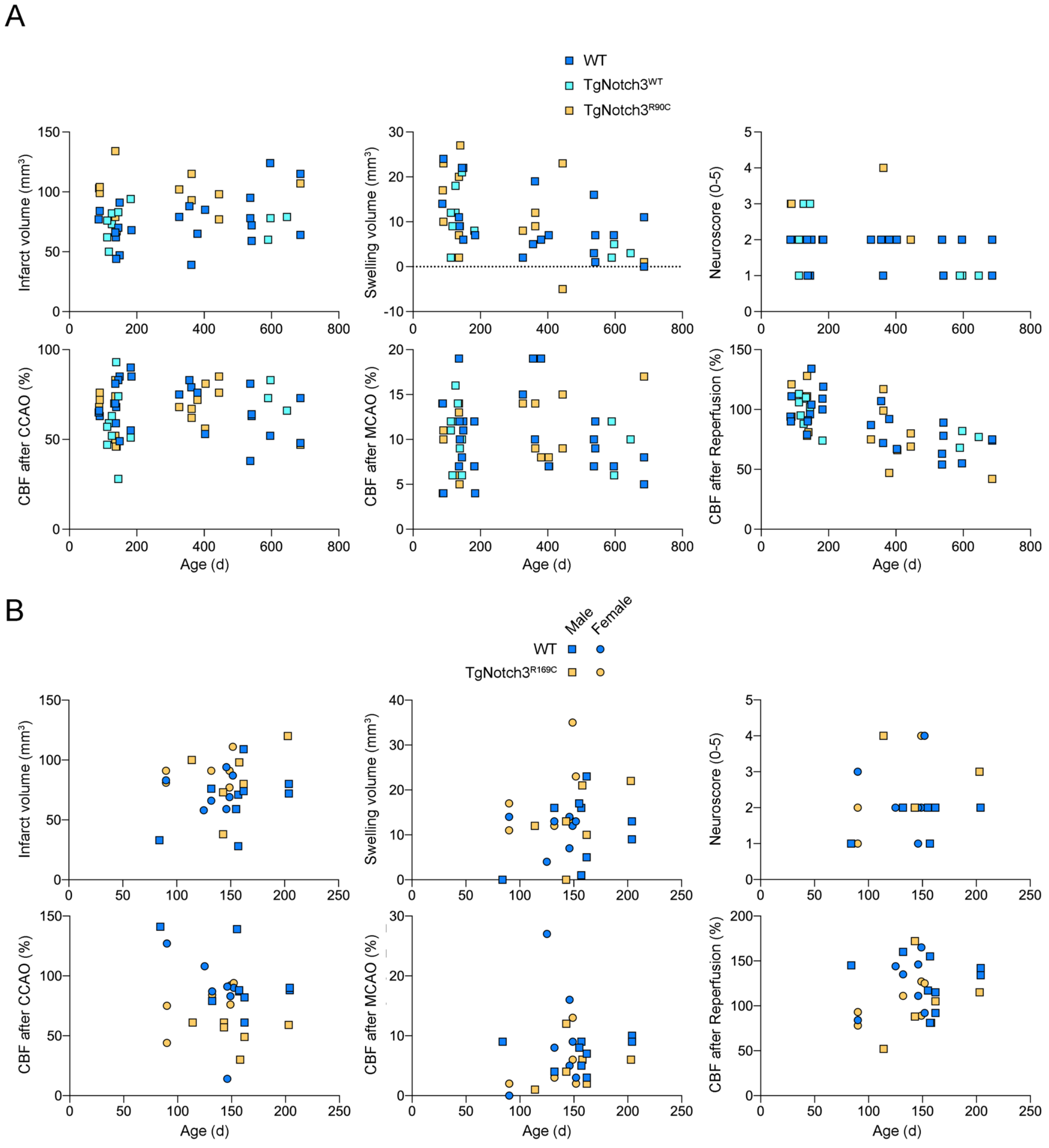

Supplementary Figure 1. Distribution of outcomes by age, genotype and sex after different stages of filament middle cerebral artery occlusion (fMCAO) in Notch3^{R90C} (A) and Notch3^{R169C} (B) cohorts. Vertical axis labels "CBF CCAO" refers to the CBF after CCA clamping, "CBF MCAO" refers to the CBF drop after filament insertion, and "CBF Reperfusion" refers to CBF after filament removal. Each data point represents one animal.

Supplementary Table 1. Systemic physiology

Experiment	Genotype	pH	pCO_2	pO_2	BP
Distal MCAO Area of CBF deficit	$TgNotch3^{WT}$	7.35±0.05	37±7	138±14	79±5
	$TgNotch3^{R90C}$	7.35±0.04	36±6	143±16	83±5
	WT	7.35±0.03	29±2	130±22	76±6
	$TgNotch3^{R169C}$	7.36±0.03	29±3	139±30	75±5
Peri-infarct SD	WT	7.37±0.02	37±3	136±27	90±4
	$TgNotch3^{R90C}$	7.38±0.02	35±4	135±20	84±11
	WT	7.35±0.02	37±3	111±10	91±4
	$TgNotch3^{R169C}$	7.35±0.04	35±4	106±7	91±8

Mean ± standard deviation.
